# Effects of Ozone Water Combined With Ultra-High Pressure on Quality and Microorganism of Catfish Fillets (*Lctalurus punctatus*) During Refrigeration

**DOI:** 10.3389/fnut.2022.880370

**Published:** 2022-07-06

**Authors:** Yuzhao Ling, Mingzhu Zhou, Yu Qiao, Guangquan Xiong, Lingyun Wei, Lan Wang, Wenjin Wu, Liu Shi, Anzi Ding, Xin Li

**Affiliations:** ^1^Key Laboratory of Cold Chain Logistics Technology for Agro-Product, Ministry of Agriculture and Rural Affairs, Institute of Agro-Products Processing and Nuclear Agricultural Technology, Hubei Academy of Agricultural Sciences, Wuhan, China; ^2^School of Environmental Ecology and Biological Engineering, Wuhan Institute of Technology, Wuhan, China; ^3^School of Bioengineering and Food, Hubei University of Technology, Wuhan, China

**Keywords:** ultra-high pressure, ozone water, catfish fillets, lipid oxidation, microbial enumeration, 16S rRNA gene sequencing

## Abstract

This study described the quality and microbial influence on ozone water (OW) and ultra-high pressure (UHP) processing alone or in combination with refrigerated catfish fillets. The analysis parameters included total volatile base nitrogen (TVBN), thiobarbituric acid reactive substances (TBARs), chromaticity, microbial enumeration, 16S rRNA gene sequencing, electronic nose (E-nose), and sensory score. The study found that compared with the control (CK), ozone water combined with ultra-high pressure (OCU) delayed the accumulation of TVBN and TBARs. The results of sensory evaluation illustrated that OCU obtained a satisfactory overall sensory acceptability. The counting results suggested that compared to CK, OCU significantly (*p* < 0.05) delayed the stack of TVC, *Enterobacteriaceae*, *Pseudomonas*, lactic acid bacteria (LAB), and hydrogen sulfide-producing bacteria (HSPB) during the storage of catfish fillets. The sequencing results reflected that the dominant were *Proteobacteria*, *Firmicutes*, *Bacteroidetes*, and *Actinobacteria* at the phylum level, and the dominant were *Acinetobacter*, *Pseudomonas*, *Lelliottia*, *Serratia*, *Shewanella*, *Yersinia*, and *Aeromonas* at the genus level. The dominant was *Acinetobacter* in initial storage, while *Pseudomonas* and *Shewanella* were in anaphase storage. Based on the TVC and TVBN, the shelf life of catfish fillets was extended by at least 3 days compared to the control. In short, the combination of ozone water and ultra-high-pressure processing is a favorable strategy to control microbial quality and delay lipid oxidation during catfish storage.

## Introduction

Channel catfish (*Lctalurus punctatus*) is the main cultured fish in the United States (1), while there are limited types of processed catfish products in China, mainly frozen fish fillets (2). The endogenous enzymatic reactions, oxidation, and microbial activities that occur after fish die could reduce sensory acceptance and nutritional value (3). Therefore, it is necessary to take timely preservation measures for catfish to maintain their economic value. Biological preservatives are natural and harmless, while their high acquisition cost and difficulty in adapting to industrialization are still critical reasons for not being commercialized (4). Certain chemical preservatives, for example, nitrite, are powerful in the preservation of meat products, but their potential carcinogenic ability keeps most consumers away (5). Based on the above, food processors are looking for harmless and efficient alternatives to extend the shelf life of catfish. Considering fresh aquatic products, non-pasteurization is the first choice. Common physical food processing technologies include electromagnetic field, cold plasma, irradiation, high pressure, high hydrostatic pressure, ozone, ultrasonic, microwave, ultraviolet, pulsed light, pulsed electric field, and shockwaves (6, 7).

Ozone is a powerful bactericide, which can quickly kill all kinds of pathogens, including protozoa, bacteria, and viruses due to its strong oxidizing property (8, 9). In the food industry, ozone water has been widely used in the disinfection of aquatic products. Pastoriza et al. (10) reported that the replacement treatment of ozone water resulted in a reduction in the total viable count (TVC) and hydrogen sulfide-producing bacteria (HSPB) compared to seawater cleaning and ice making. However, ozone water alone is not enough to effectively inhibit certain spoilage bacteria for example *Pseudomonas*. Tachikawa et al. (11) found that *Pseudomonas fluorescens* and *Pseudomonas aeruginosa* in biofilms were more resistant to ozone damage than suspension cells. Therefore, hurdle technology is used as a strategy to enhance the bactericidal effectiveness. Ultra-high pressure processing (UHP), also known as ultra-high hydrostatic pressure processing, is considered to be an emerging and promising physical alternative to heat sterilization (12). UHP (usually 100–800 MPa) has been proven to inactivate a variety of microbes and endogenous enzymes while retaining the sensory properties and nutritional value of food (6, 13–15). Specifically, UHP can cause membrane damage and increase the permeability of the cell membrane, which is the main reason for the destruction of microbes. In addition, the destruction of organelles and genetic material may also be critical reasons (16). In the food industry, UHP has been widely used as a pasteurization step after food packaging (17). Ye et al. (18) reported the effect of high-pressure treatment (300 MPa/20 min) on crab meat during storage at 4°C, and the results showed that the TVC (5.71 log_10_ CFU/g) on the 8th day was still lower than the limit value (6 log_10_ CFU/g) recommended by the International Committee of Microbiological Specializations on Food (ICMSF). But expensive equipment, localized packaging options, limited inactivation of spores, cooked appearance, and lipid oxidation are major restrictions to the widespread application of ultra-high pressure (13, 16).

Microbial activity and lipid oxidation are the main factors that limit the shelf life of fish (4). Two emerging technologies, ozone water and ultra-high pressure are considered harmless, effective, and economical methods, and are widely used in food technology. Ozone water or ultra-high pressure alone used to effectively control food spoilage has been widely reported. However, there are rare reports to probe the effects of ozone water combined with ultra-high pressure on refrigerated catfish for all we know. In this study, we checked the changes in the quality of refrigerated catfish fillets treated by single or combined ultra-high pressure through physicochemical parameters [total volatile base nitrogen (TVBN), thiobarbituric acid reactive substances (TBARs), chromaticity, and electronic nose], microbial analysis (microbial plate count and next-generation sequencing), and sensory evaluation.

## Materials and Methods

### Fillet Samples Preparation

The live catfish (mean weight and body length were about 2.0 ± 0.5 kg and 50 ± 5.0 cm, respectively) purchased from a local Wushang supermarket (Hongshan District, Wuhan, China), were transported to the laboratory in 1 h. Cleaned catfish were slaughtered, and then the back muscles were cut into small blocks (mean weight of about 50 g). All untreated fish fillets were divided into four groups as follows: ultrapure water immersion for 10 min set as the control (lot CK), 13.28 mg/L ozone water immersion for 10 min (lot OW), 200 MPa high-pressure treatment for 10 min (lot UHP), and first 13.28 mg/L ozone water immersion for 10 min followed by 200 MPa high-pressure treatment for 10 min (lot OCU). In detail, a gas–liquid mixer (HPSJ-25, Wuhan, China) combined with an ozone generator (GCQJ-1-3, Wuhan, China) was used to produce ozone water, and the flow rate was adjusted to change the concentration of ozone water. The soaked fish fillets were packed in cooking bags, and then the cooking bags were placed in a vacuum sealer (DZD-400/S, Tengtong Co. Ltd., Nantong, China). In addition, the packaged fillets from UHP and OCU were sent to an ultra-high pressure processing machine (HPPL2-600MPa/2L, Huataisenmiao Inc., Tianjin, China). All packaged fillets were stored at 4°C, and the physicochemical, microbiological, and sensory parameters of the catfish fillets were evaluated on the 0th, 3rd, 6th, 9th, and 12th days, respectively (unless otherwise specified).

### Measurement of Total Volatile Base Nitrogen

Accurately 10.0 g minced catfish meats and 90.0 ml of 7.5% (*w/v*) trichloroacetic acid solution were homogenized at 5,000 rpm for 1 min, and then the mixture was filtered with a GE Whatman medium-speed qualitative filter paper after centrifuging at 5,000 rpm for 10 min at 4°C. The semi-micro-quantitative nitrogen method was used to determine TVBN in catfish fillets according to the fore method (19) with a slight modification. Concretely, 5 ml of obtained filtrate and 5 ml of 1% (*w/v*) magnesium oxide were boiled in a reaction chamber for 6 min, and the receiving parts were 10 ml of 2% (*w/v*) boric acid absorption solution and a few drops of indicator (methyl red and bromocresol green at a ratio of 1:5). After the reaction, 0.01 mol/L hydrochloric acid standard solution was used to turn the indicator blue–violet. TVBN of the reactant was converted by the volume of hydrochloric acid standard solution, and its value was expressed as mg N per 100 g of catfish fillets. Each catfish fillet was measured in triplicate.

### Measurement of Thiobarbituric Acid Reactive Substances

Accurately 5 g of minced catfish muscles were homogenized with 45 ml of 20% (*w/v*) trichloroacetic acid solution. After standing for 30 min, the mixture was centrifuged at 5,000 rpm for 10 min at 4°C. The centrifugal liquid obtained was collected after passing through a GE Whatman medium-speed qualitative filter paper. About 5 ml of supernatant and 5 ml of 0.02 mol/L thiobarbituric acid (TBA) were seethed for 30 min. The reacting substance was measured at 532 nm using a UV-vis spectrophotometer (UV-3802, Unico Instrument Co. Ltd., China) after cooling in an ice bath. 1,1,3,3-Tetraethoxypropane was used in the preparation of the standard curve, and then TBARs was expressed as mg malondialdehyde (MDA) per kilogram of catfish fillets. Each catfish fillet was measured in triplicate.

### Examination of Chromaticity

The L* (representing brightness value), a* (representing red or green value), and b* (representing yellow or blue value) of catfish fillets with a thickness of 40 mm were measured by a portable colorimeter (CR-400, Konica Minolta Inc., Japan), and the colorimeter was corrected by a white standard board (L* = 85.6, a* = 0.3162, b* = 0.3238) before testing. The chromaticity of three catfish fillets was measured in triplicate, and random testing parts of each catfish fillet were selected. The whiteness and chromatic aberration (ΔE) were expressed with the following equation (20, 21):


$$\text{Whiteness}=100-\sqrt{{\left(100-\text{L}\right)}^{2}+{\text{a}}^{2}+{\text{b}}^{2}}$$

$$\Delta \,\text{E}=\sqrt{{\left(\Delta \,L\right)}^{2}+{\left(\Delta \,a\right)}^{2}+{\left(\Delta \,b\right)}^{2}}$$

Where ΔL*, Δa*, and Δb* represent the aberration in the L*, a*, and b* values of different processing and raw catfish fillets.

### Microbiological Evaluation

#### Microbial Enumeration

Precisely 10.0 g of chopped catfish muscles was thoroughly mixed with 90 ml of sterile 0.85% (*w/v*) saline and sterile grass beads to obtain a sample suspension. Then, 1 ml of the suspension was taken at an appropriate dilution to a petri dish containing approximately 20 ml of agar medium, while gently rotating the petri dish to fully mix the bacterial solution and the culture medium. After the agar medium had solidified, the plates were placed upside down into an artificial constant temperature incubator (HPX-9082 MBE, Boxun Inc., China). These procedures were completed in the ultra-clean workbench (HFsafe-1200LC, Lishen Inc., China). The strategy of microbial selective counting was customized based on the previous studies (22, 23): (a) the total viable count (TVC) was cultivated on Plate Count Agar (Hopebio, China) at 30°C for 3 days, (b) *Enterobacteriaceae* were incubated on Violet Red Bile Glucose Agar (Hopebio, China) at 30°C for 2 days, (c) *Pseudomonas* were cultivated on Cetrimide Fucidin Cephaloridine Agar (Hopebio, China) added with selective supplements at 30°C for 3 days, (d) lactic acid bacteria (LAB) were incubated on De Man Rogosa Sharpe Agar (Hopebio, China) at 30°C for 3 days, and (e) hydrogen sulfide-producing bacteria (HSPB) were incubated on Triple Sugar Iron Agar (Hopebio, China) at 30°C for 3 days (the production of black colonies). Two fish fillets from each treatment were respectively used for microbial enumeration. The counting results from the plates with 30–300 colonies were converted to the colony-forming units (CFU) and expressed in the form of log_10_ CFU/g.

#### Microbial Diversity

The 0th and 6th days of CK were used for sequencing analysis given the previously obtained results of the TVC threshold. Similarly, OW and UHP: the 0th and 9th days; OCU: the 0th and 12th days. The cotton swabs contaminated with the contents were stored at –80°C and then sent to Meiji Biomedical Technology Co. Ltd. (Shanghai, China) for microbial sequencing. The QIAamp^®^ DNA Tool Mini Kit was used to extract bacterial DNA from swabs, and 2.0% agarose gel electrophoresis was used to determine the DNA quality. Universal primer 1 (338-F) and primer 2 (806-R) were used to amplify the V3-V4 region of the 16S rRNA gene according to Zhang et al. (24) with appropriate modification. The PCR mix included TE buffer, 10 ng of DNA template, 4 μl of 5 × FastPfu buffer, 0.4 μl of FastPfu DNA polymerase, 0.8 μl of primer 1, 0.8 μl of primer 2, and 2 μl of dNTP (2.5 mM). The PCR amplification procedures are listed as follows: 94°C for 2 min, and followed by 30 cycles: degeneration at 94°C for 1 min, renaturation at 55°C for 40 s, elongation at 72°C for 40 s, and finally, extension at 72°C for 10 min. The amplification process was completed in the ABI GeneAmp^®^ 9700 PCR sprint. The concentration of amplified DNA was identified by 2.0% agarose gel electrophoresis, and the TruSeq^®^ Nano DNA LT Sample Prep Kit (Illumina Inc., San Diego, CA, United States) was used to purify the amplified DNA, and then the sequencing library was established.

The Illumina HiSeq platform (Beijing Novogene Bioinformation Science and Technology Co. Ltd., China) was used to sequence the amplified DNA. The Flash (v1.2.11) software was used to splice, denoise, merge, and non-chimeric the sequences to obtain high-quality sequences. The Uparse (v7.0.1090) software clustered high-quality sequences into the same operational taxon units (OTU) with a similarity of 97%. OTU could be converted into a genus or a phylum. To obtain the species classification information corresponding to each OTU, the silva132/16S_bacteria database was used for taxonomic comparison of species classification, and Ribosomal Database Project (RDP) classifier (v2.11). The confidence percentage of species classification was set as 70%. This procedure was carried out on the free online platform from Shanghai Majorbio Bio-pharm Technology Co. Ltd. Plotting was based on data with software R (v3.3.1), including Principal Co-ordinates Analysis (PCoA), Barplot, and Heat-map to visualize bacteria community.

### Electronic Nose Analysis

The electronic nose (E-Nose) (PEN3, AIRSENSE Co. Ltd., Germany) was used to analyze the odor difference of the crayfish samples. About 2.0 g of shredded catfish muscles and 4 ml of saturated saline were placed in a 20.0 ml headspace and sealed with an E-Z Crimper (Huifen Co. Ltd., China). The headspace bottle was placed in a 45°C water bath to equilibrate for 2 min and then analyzed in the E-nose system. The test parameters were as follows: flush time and measurement time were set as 100 and 120 s, respectively, and chamber flow was set as 600 ml/min. The E-nose system consists of 10 metal oxide sensors, which are sensitive to different volatile components. The response values of the sensor from 115 to 119 s were used to visualize the odor difference between the samples, and this procedure was completed in the software WinMuster built into the E-nose system. The samples from day 0 and day 12 were used for the E-nose test, and each sample was tested in triplicate.

### Sensory Evaluation

Sensory evaluation was evaluated through four indexes: smell, color, muscle tissue, and elasticity, and slightly modified by Li et al. (25). Each indicator included four summary descriptive words based on sensory. The descriptors respectively represented four excellent quality levels (representing 8–9 score), good (representing 6–7 score), average (representing 4–5 score), and poor (representing 2–3 score). Five experienced food professionals (three males and two females) aged 22–26 in the laboratory scored the descriptors, and the results were averaged.

### Statistical Analysis

Excel 2010 (Microsoft Corporation, United States) was used to calculate data, and Origin 9.4 (Origin Lab Corporation, Northampton, MA, United States) was used to form the chart. SPSS 18.0 for Windows (SPSS Inc., Chicago, IL, United States) was used for multiple comparisons (one-way analysis of variance with the Duncan method) between data, and a confidence interval of 95% (*p* < 0.05) was set as the significance level. The results were expressed as means ± standard deviation (SD).

## Results and Discussion

### Total Volatile Base Nitrogen

**FIGURE 1:**
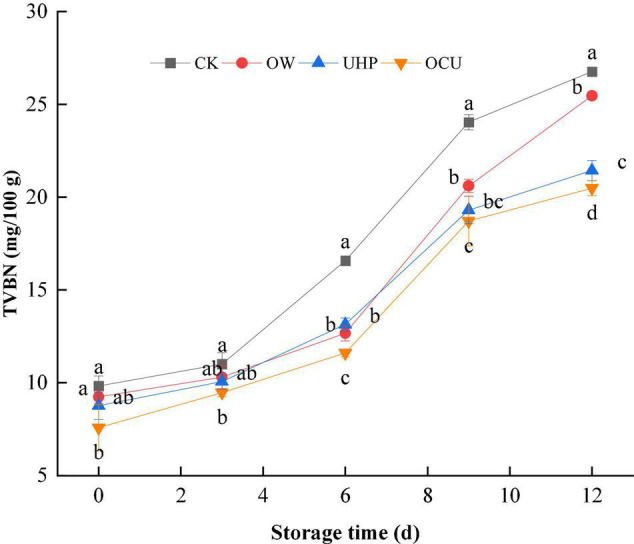
Changes in total volatile basic nitrogen (TVBN) of refrigerated catfish fillets. Error bars were derived from the standard deviation of means. Significant differences (*p* < 0.05) were expressed as alphabets (a, b, c, and d) in different treatments at the same storage time, respectively. CK, the control; OW, ozone water; UHP, ultra-high pressure; OCU, ozone water combined with ultra-high pressure.

As shown in Figure 1, the TVC of the different treatments gradually increased with the prolonged storage. The TVBN value of all treatments was less than 12 mg/100 g on day 0. After that, the TVBN of CK increased rapidly and exceeded the freshwater fish threshold (20 mg/100 g) defined by the Chinese National Food Safety Standard (GB 2733–2015) on the 9th day. The combined treatment did not reach this spoilage point until the 12th day, which prolonged the shelf life by at least 3 days compared with the control. In brief, the TVBN of OCU was significantly (*p* < 0.05) lower than that of CK during the entire storage, which suggested that ozone water combined with ultra-high pressure could effectively reduce the accumulation of TVBN produced by spoilage bacteria and endogenous protein enzymes. Similarly, studies reported that TVBN of red mullet and coho salmon was reduced to varying degrees at different pressure levels compared to unpressured treatments (26, 27). On the other hand, we observed that the TVBN of UHP was lower than OW in general, and this phenomenon was more pronounced in anaphase storage (9–12 days).

### Thiobarbituric Acid Reactive Substances

**FIGURE 2:**
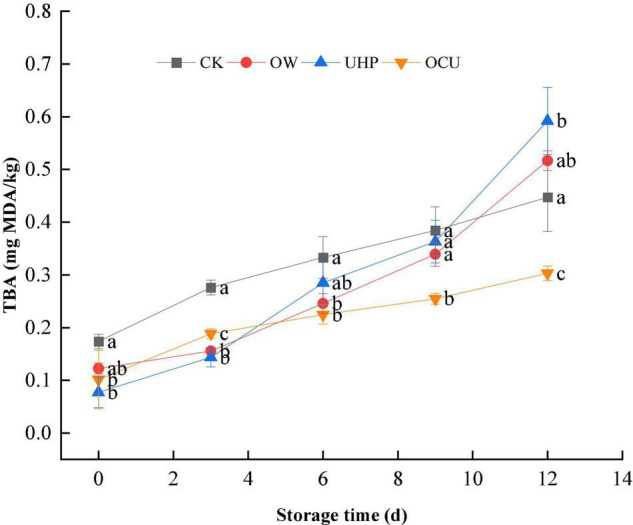
Changes in thiobarbituric acid reactive substances (TBARs) in refrigerated catfish fillets. Error bars were derived from the standard deviation of means. Significant differences (*p* < 0.05) were expressed as alphabets (a, b, c, and d) in different treatments at the same storage time, respectively. CK, the control; OW, ozone water; UHP, ultra-high pressure; OCU, ozone water combined with ultra-high pressure.

The TBARs monitoring of various secondary oxidation products is a valuable parameter to characterize lipid oxidative rancidity. As shown in Figure 2, with the storage prolongation, the TBARs of CK, OW, UHP, and OCU increased gradually. Previous studies have found that ozone water or ultra-high pressure processing could lead to increased TBARs (28–32). The TBARs of the three processing methods were lower than that of the control in early storage. The TBARs of OCU were significantly (*p* < 0.05) lower than that of CK during the entire storage, which suggested that ozone water combined with ultra-high pressure could effectively restrain rancidity. Pressurization could prevent the development of lipid oxidation by inhibiting the activity of endogenous enzymes (lipoxygenase, peroxidase, etc.) (15). Torres et al. (33) found that high-pressure treatment partially inhibited the development of lipid oxidation in Atlantic horse mackerel during frozen storage. The TBARs of OCU that we observed may be due to the better enzymatic inactivation effect of the combined treatment than either UHP or OW alone. The milder pressurization level used in this study (pressure is 200 MPa, and the holding time is only 10 min) may also be a reason for not complying with the UHP effect on lipid oxidation. The explanation of these phenomena was also classified as the differences in the composition and content of lipids in the species (34), the position of muscle containing heme proteins, and the pressurized level (such as pressure and duration) (31, 35).

### Chromaticity

**FIGURE 3:**
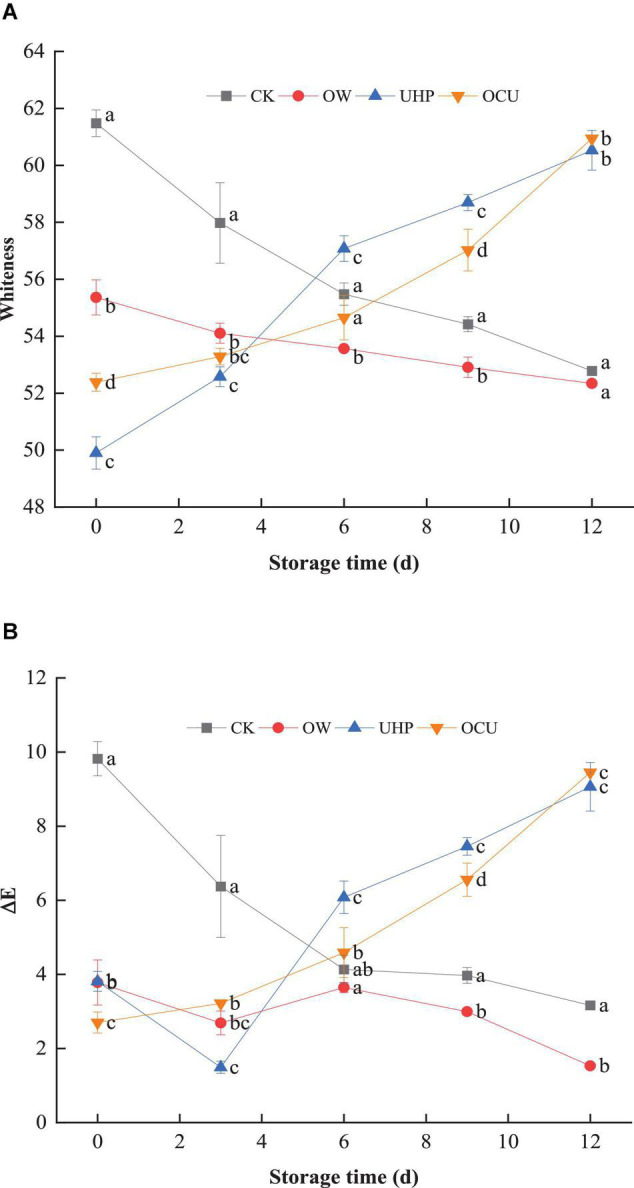
Panels **(A,B)** were respectively whiteness and chromatic aberration of refrigerated catfish fillets. Error bars were derived from the standard deviation of means. Significant differences (*p* < 0.05) were expressed as alphabets (a, b, c, and d) in different treatments at the same storage time, respectively. CK, the control; OW, ozone water; UHP, ultra-high pressure; OCU, ozone water combined with ultra-high pressure.

The whiteness (Figure 3A) of OW was significantly (*p* < 0.05) lower than that of CK until the storage destination. In general, individual ozonation results in increased whiteness (36, 37). Ozone water causes the color difference of species. de Mendonça Silva et al. (32) found that ozone water processing did not affect the color of Nile tilapia fillets. These discrepancies may be due to factors such as the biological conditions of the species, storage conditions, and the treated load they receive. In addition, the whiteness of OCU was lower than that of CK until the 6th day. In detail, the whiteness of CK decreased from 61.48 on day 0 to 55.48 on day 6, while the whiteness of OCU increased from 52.38 on day 0 to 54.65 on day 6, indicating that OCU reduced the bleaching of the fillets compared to CK until storage middle. Subsequently, storage was prolonged, contrary to the serial decline of CK, the whiteness of UHP and OCU continued to rise. The results we observed reflected that catfish fillets treated with ultra-high pressure developed a cooked color, which may be attributed to the degeneration of the myofibrils and sarcoplasmic proteins that lead to changes in the surface of the meat (28). Similarly, studies have reported that high pressure processing contributed to the cooked appearance of meat (38, 39). Globin denaturation, oxidation of myoglobin, the disintegration of myofibrillar protein, lack of active pigments, and protein coagulation were also considered to be contributing factors to the whitish color (30, 40–43). And pressurization intensity could also affect color differences. Ragazzo-Sanchez et al. (44) reported that 600 MPa pressure treatment for 180 s did not affect the color of the shrimp paste samples. The converted ΔE (Figure 3B) and whiteness displayed the same trend. The L*, a*, and b* of raw catfish fillets were 51.74, 2.35, and 0.98, respectively (data not shown). Moreover, OCU contributed to the minimum of ΔE on day 0 (*p* < 0.05), and its increase was proportional to the prolonged storage. See the Supplementary Material for the actual photos of refrigerated catfish fillets.

### Microbial Count

**FIGURE 4:**
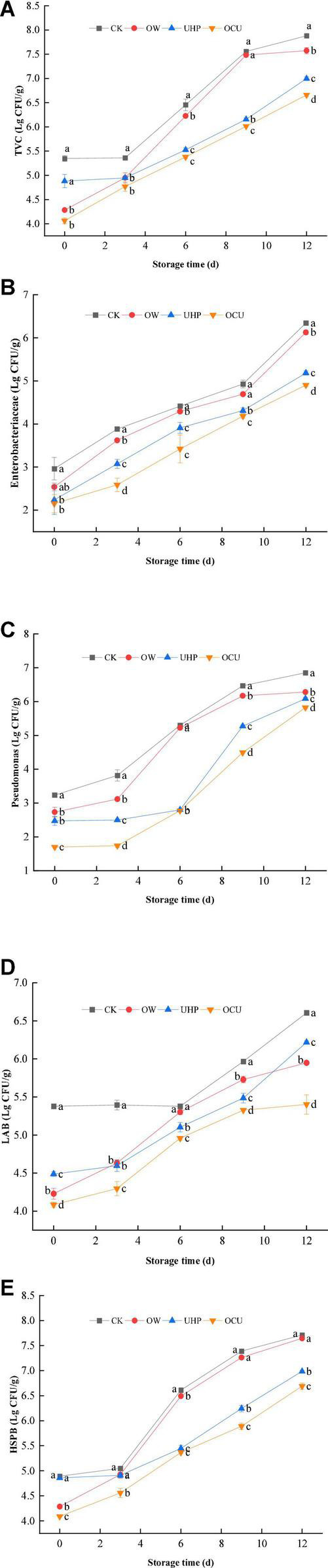
Microbial count of refrigerated catfish fillets, including **(A)** total viable count (TVC), **(B)** *Enterobacteriaceae*, **(C)** *Pseudomonas*, **(D)** lactic acid bacteria (LAB), and **(E)** hydrogen sulfide-producing bacteria (HSPB). Error bars were derived from the standard deviation of means. Significant differences (*p* < 0.05) were expressed as alphabets (a, b, c, and d) in different treatments at the same storage time, respectively. CK, the control; OW, ozone water; UHP, ultra-high pressure; OCU, ozone water combined with ultra-high pressure.

As shown in Figure 4A, the TVC of different processing was positively correlated with prolonged storage. Specifically, TVC of OW, UHP, and OCU was 4.28, 4.88, and 4.06 log_10_ CFU/g at day 0, respectively, which were all lower than 5.35 log_10_ CFU/g of CK. The initial bacterial count of CK was relatively high and exceeded the threshold (6 log_10_ CFU/g) stipulated in Al–Dagal et al. (45) on the 6th day. The OCU did not reach this spoilage point until the 9th day, which prolonged the shelf life by at least 3 days compared with the control. The microbial activity was also evaluated by chemical parameters, for example, TVBN (27). Dramatically, the TVC results we observed were consistent with the formerly described TVBN. The latest evidence confirmed that there was a significant correlation between alkaline volatile nitrogen and microbial proliferation (46). In brief, TVC of OCU was significantly (*p* < 0.05) lower than that of CK during the entire storage, which suggested that ozone water combined with ultra-high pressure could effectively reduce the accumulation of bacteria and fungi. On the other hand, we observed that the microbial loading of UHP was lower than OW in general, and this phenomenon was more significant (*p* < 0.05) in 6 –12 days. Zhao et al. (47) found that *Toona sinensis* treated with UHP reduced more TVC compared with ozonation. The lower initial microbial load of OW may be attributed to the sublethal damage to the cells. After the harsh stress is eliminated, cell metabolism can be partially or completely repaired (44, 48). The explanation of OCU may be attributed to ultra-high pressure further ruptured the cells damaged by oxidation, resulting in irreversible degeneration. Then, we emphasized the potential of ozone water combined with ultra-high pressure to control the microbial quality of catfish fillets.

*Enterobacteriaceae* (Figure 4B), *Pseudomonas* (Figure 4C), LAB (Figure 4D), and HSPB (Figure 4E) gradually increased during storage. Both *Enterobacteriaceae* and *Pseudomonas* from combined treatment were significantly (*p* < 0.05) less than from the control during storage. In summary, the combined processing postponed the accumulation of the main genera of *Proteobacteria* in the entire storage. Spoilage microorganisms such as *Pseudomonas* and *Shewanella* could metabolize free amino acids in the muscles to produce volatile nitrogen compounds (49), resulting in unacceptable sensory quality. The counting results showed that *Pseudomonas* has the potential to survive in ozone water based on a large increase of OW in mid-to-late storage. And preceding reports also stated that *Pseudomonas* has the advantage of tolerating a fairly high dose of ozone water (50). The results we found suggested that UHP could effectively rupture the cell membrane of *Pseudomonas*. Similarly, ultra-high pressure reduced the initial load of *Pseudomonas* (27). Moreover, Rivas-Canedo et al. (51) reported that the presence of some gram-negative bacteria such as *Pseudomonas* and *Aeromonas* was directly related to accelerated lipid oxidation. Our reduced *Pseudomonas* may also contribute to the reduced TBARs as described earlier. The oxygen-free environment caused by vacuum is conducive to facultative anaerobes propagation, which also explained that certain LAB can become dominant bacteria in later storage (52). In short, the LAB of ultra-high-pressure treatments was significantly (*p* < 0.05) reduced compared with the control. The study reported that the same pressure (200 MPa) treatment can effectively reduce the LAB of salmon fillets during refrigeration, no matter at the beginning or end of storage (53). Compared to the control, the LAB of beef and chicken breast pressure-treated at 400 MPa/10 min decreased significantly (51). H_2_S is described as a strong characteristic spoilage odor of “spoiled eggs.” In general, the trend of HSPB was not much different from that of TVC. Ozone water lethality to HSPB during entire storage was mild on the whole except for the significant (*p* < 0.05) decline on day 6. The low initial bacterial load of OW was explained as ozone water processing may cause sublethal bacterial damage, and this damage gradually recovers as prolonged storage. Compared with OW, OCU significantly (*p* < 0.05) reduced the load of HSPB during storage, which also indicated that the inactivation efficacy of the combined treatment mainly came from ultra-high pressure. A previous study found that *Shewanella* (a representative genus producing hydrogen sulfide) achieved varying degrees of reduction through ultra-high pressure levels (27). In brief, the combination of ozone water and ultra-high-pressure processing has achieved a significant bacterial inactivation amount.

### Microbial Diversity

**FIGURE 5:**
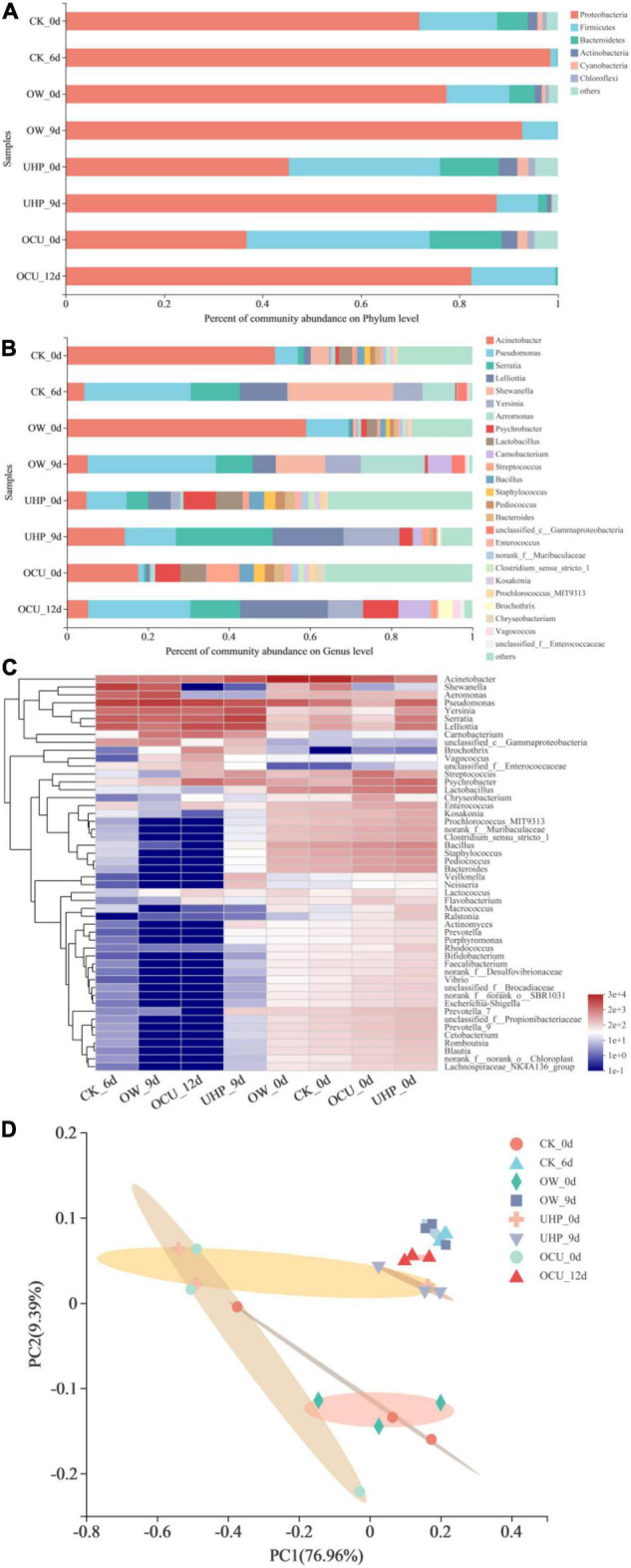
The relative abundance of bacterial community phylum **(A)** and genus **(B)** level in refrigerated catfish fillets. Among panel **(B)**, genera with a relative abundance of less than 1% (all samples) were classified as “other”; panel **(C)** was bacterial community analysis at the genus level, presented in the form of a Heat-map (readers can choose the online version of this article to refer to the colors in the Heat-map); panel **(D)** was principal co-ordinates analysis (PCoA) at the species level. CK, the control; OW, ozone water; UHP, ultra-high pressure; OCU, ozone water combined with ultra-high pressure.

Most OTU belonged to four bacteria phyla (Figure 5A), namely *Proteobacteria*, *Firmicutes*, *Bacteroidetes*, and *Actinobacteria*. Based on the results on day 0, the relative abundance of the most abundant phylum *Proteobacteria* from UHP and OCU decreased compared to CK, but excluding OW. In detail, the relative abundance of CK, OW, UHP, and OCU was 60.03, 68.82, 19.85, and 22.16%, respectively. The results emphasized that ultra-high pressure has better bacteria reduction ability than ozone water, which complied with our previous results of TVBN and TVC. Generally, compared with gram-negative bacteria, gram-positive bacteria survived more due to their thicker cell walls with 40 layers of peptidoglycan and their ability to resist ultra-high pressure (44).

A total of twenty-five of relative abundance value >1% (at least 1 sample) was identified at the genus level (Figure 5B). OTU of most genera increased with prolonged storage. On day 0, the combined processing significantly reduced the relative abundance of *Acinetobacter* and *Pseudomonas* belonging to the phylum *Proteobacteria*, while the ozone water processing alone stimulated the vitality of genera. Concretely, *Acinetobacter* and *Pseudomonas* were reduced by 33.73 and 4.26%, respectively. In this work, *Acinetobacter* showed high relative abundance in initial storage, while *Pseudomonas* became the most dominant bacteria in anaphase storage, which may be attributed to competition between species. A previous study reported that *Acinetobacter*, which dominated fresh fish, was replaced by *Pseudomonas* or *Aeromonas* under different treatments (54). In addition, the abundances of *Lelliottia*, *Serratia*, *Shewanella*, and *Yersinia* belonging to the phylum *Proteobacteria* were less than 1% (all samples) after combined processing (OCU). *Serratia* belonging to *Enterobacteriaceae* was widely considered to be typical conditional rot-causing bacteria (55, 56). Belletti et al. (57) expressed the fact that *Serratia* was affected by high pressure. In short, the results at the genus level corresponded to those at the phylum level as well as the *Enterobacteriaceae* count. At the same time, these phenomena indicated that ozone water combined with ultra-high pressure effectively deactivated *Enterobacteriaceae* of the *Proteobacteria*. *Shewanella* belonging to *Proteobacteria* was recognized as the main contributor to hydrogen sulfide during the storage of aquatic products (58). As for *Shewanella*, its relative abundance was reduced in the ultra-high-pressure treatments (UHP and OCU) compared to the control during storage, and the effective reduction of *Shewanella* in ultra-high pressure followed the previous HSPB count result. On the other hand, certain gram-positive bacteria such as *Lactobacillus*, *Bacillus*, *Staphylococcus*, *Pediococcus*, and *Enterococcus* were insensitive to ultra-high-pressure processing given increased relative abundance in initial storage. Due to the presence of thick film or endospores, the gram-positive bacteria can effectively withstand ultra-high pressure (48). The study reported that the gram-negative bacteria group and coliforms decreased more than TVC and LAB under the same pressure level treatment (51).

The top fifty microbial genera in total abundance emerged in the Heat-map (Figure 5C). The colors of most genera that characterize relative abundance had obvious differences between the early and late storages, which indicated that the dominant microbiota had changed. The Principal Co-ordinates Analysis (PCoA) plot showing species clustering is exhibited in Figure 5D. The PCoA visualized the differences in microbial species between different processing methods. The first principal component (PC1) and the second principal component (PC2) were 44.33 and 20.08%, respectively. In initial storage, OTU were clustered into two areas, one formed between UHP and OCU, and the other between CK and OW. The species composition similarity between UHP and OCU was attributed to the inactivated effects of ultra-high pressure on microbial communities. In addition, OW was closest to CK due to the limitation of the bactericidal effect of ozone water. Consistent with the previous microbial enumeration results, the inactivation of ultra-high pressure was more recognized than that of ozone water.

### Electronic Nose

**FIGURE 6:**
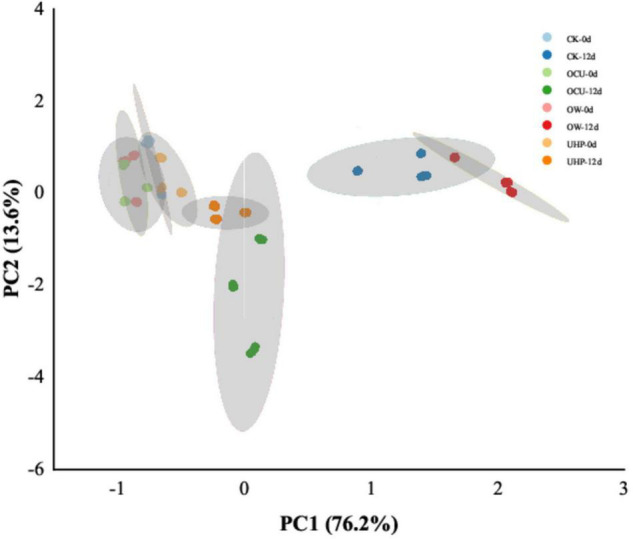
Principal components analysis (PCA) of electronic nose (E-nose) odor clustering in refrigerated catfish fillets. CK, the control; OW, ozone water; UHP, ultra-high pressure; OCU, ozone water combined with ultra-high pressure.

Electronic nose technology was considered a simple and fast parameter for characterizing complex odors in food samples (59). Figure 6 is principal components analysis (PCA), showing the odor clustering of refrigerated catfish fillets from different processing. The sample variance of PC1 and PC2 was 96.63 and 2.33%, respectively, which almost characterized the volatile odor components of all catfish fillets. An overlap area was created between CK, OW, UHP, and OCU due to fresh samples, which suggested that the contribution of the samples with several processing methods to the collection of volatile odors was almost equal in the initial storage. The obvious odor difference between the samples was reflected in the anaphase storage. The interlaced area of CK and OW may be attributed to the weaker bactericidal effect of ozone water. There was a great overlap area between UHP and OCU, indicating that the odor properties of the two were approximately equal. Generally, volatile odors are mainly derived from metabolites produced by protein degradation and fat oxidation during storage as well as microbial catabolites (18). The odor distance of the ultra-high-pressure processing (UHP and OCU) on day 12 was closer to that of the fresh samples, probably because UHP inhibited the growth of putrefying bacteria and reduced the generation of some spoilage odors, which still requires further study to confirm.

### Sensory Score

**FIGURE 7:**
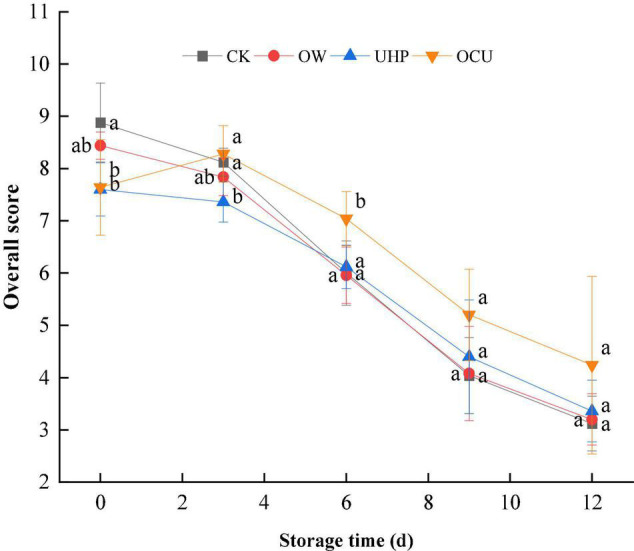
Overall sensory score of refrigerated catfish fillets. CK, the control; OW, ozone water; UHP, ultra-high pressure; OCU, ozone water combined with ultra-high pressure. Significant differences (*p* < 0.05) were expressed as lowercase alphabets in different treatments at the same storage time, respectively. CK, the control; OW, ozone water; UHP, ultra-high pressure; OCU, ozone water combined with ultra-high pressure.

Figure 7 shows the overall sensory acceptability of the samples during storage. The sensory scores of all samples decreased with prolonged storage. The scores of UHP and OCU on day 0 were 7.60 and 7.64, respectively, which attribute to cooked color caused by ultra-high-pressure treatment. From the 3rd day, OCU consistently maintained the highest overall acceptability compared with other samples. A score of 5 could be considered a threshold for not being accepted by consumers (60). CK (4.04 score), OW (4.08 score), and UHP (4.40 score) all reached levels of sensory rejection on day 9, while OCU (4.24) reached this threshold on day 12. Sensory rejection is related to microbial consumption of meat nutrients (61). The reason for the high sensory score of OCU in our results can be considered to be the synergistic inactivation effect of ozone water combined with ultra-high pressure. These sensory scores followed the changes in E-nose, indicating that the combined treatment effectively improved the sensory quality of refrigerated catfish fillets.

## Conclusion

This study suggested that ozone water combined with ultra-high pressure effectively decreased TVBN value and microbial load in catfish fillets during refrigeration. All analytical results suggested that the combined treatment inhibited catfish fillet spoilage, especially showing a significant reduction of *Enterobacteriaceae*, *Pseudomonas*, LAB, and HSPB. On the other hand, 16S rRNA gene sequencing analysis showed that the dominant bacteria in fresh catfish fillets were *Acinetobacter*, while the dominant bacteria in spoiled catfish fillets were mainly *Pseudomonas* and *Shewanella*. Further work needs to be performed to better understand the potential synergistic active mechanisms of ozone water and ultra-high pressure on spoilage bacteria, and to apply this method to extend the shelf life of catfish fillets during refrigeration.

## Data Availability Statement

The data presented in this study are deposited in the National Center for Biotechnology Information (NCBI) repository, accession number PRJNA837024.

## Author Contributions

YL: experiments, statistical analysis of data, visualization, and manuscript writing and revision. MZ: experiments and manuscript revision. YQ: experimental design and conceptualization. GX: project proposal and research methodology. LWe: supervision. LWa, WW, LS, AD, and XL: review and editing. All authors contributed to the article and approved the submitted version.

## Conflict of Interest

The authors declare that the research was conducted in the absence of any commercial or financial relationships that could be construed as a potential conflict of interest.

## Publisher’s Note

All claims expressed in this article are solely those of the authors and do not necessarily represent those of their affiliated organizations, or those of the publisher, the editors and the reviewers. Any product that may be evaluated in this article, or claim that may be made by its manufacturer, is not guaranteed or endorsed by the publisher.

## Abbreviations

OW: ozone water
UHP: ultra-high pressure
OCU: ozone water combined with ultra-high pressure
TVBN: total volatile basic nitrogen
TBARs: thiobarbituric acid reactive substances
MDA: malondialdehyde
Δ E: chromatic aberration
TVC: total viable count
LAB: lactic acid bacteria
HSPB: hydrogen sulfide-producing bacteria
OTU: operational taxon units
PCoA: principal co-ordinates analysis
E-nose: electronic nose
PCA: principal components analysis.
