# Supplementary figures and images for: Effects of Ozone Water Combined With Ultra-High Pressure on Quality and Microorganism of Catfish Fillets (Lctalurus punctatus) During Refrigeration
Source: Front Nutr. 2022 Jul 6;9:880370. doi: 10.3389/fnut.2022.880370 (PMC9298495; doi:10.3389/fnut.2022.880370)

**Actual photos of refrigerated channel catfish fillets**

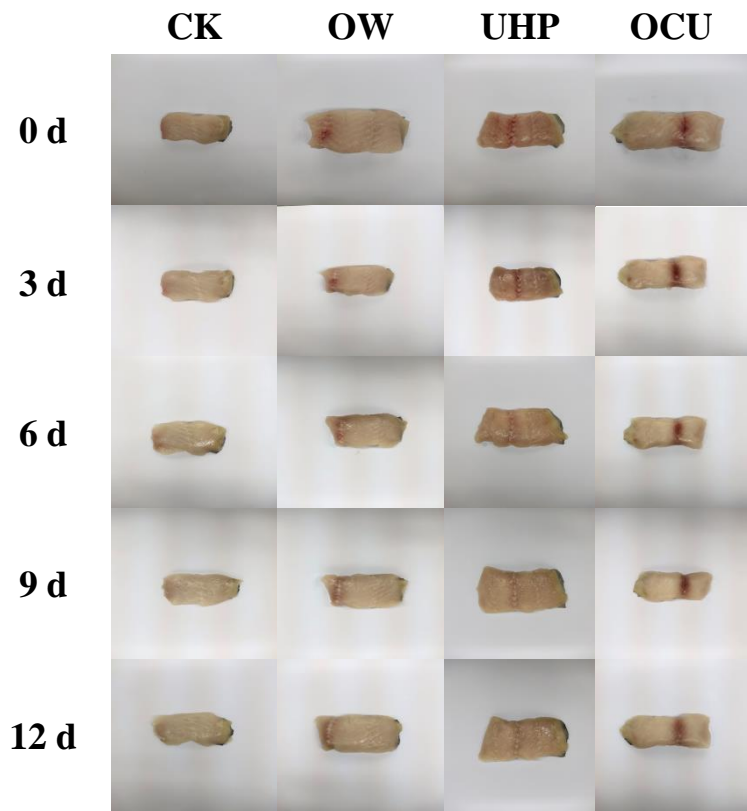

Supplement: Supplementary file 1 [file Image_1.pdf]
